# Robot-Assisted Pyelolithotomy in Pelvic Kidney

**DOI:** 10.3390/jcm13247727

**Published:** 2024-12-18

**Authors:** Husny Mahmud, Asaf Shvero, Nir Kleinmann, Zohar A. Dotan, Dorit E. Zilberman

**Affiliations:** 1 Department of Urology, Chaim Sheba Medical Center, Tel-Hashomer, Ramat Gan 5262000, Israel; 2 Sackler Faculty of Medicine, Tel Aviv University, Tel Aviv 6997801, Israel

**Keywords:** da-Vinci robotic system, nephrolithiasis, pyelolithotomy, pelvic kidney

## Abstract

**Background/Objectives:** Pelvic kidney is a congenital anomaly characterized by the kidney’s failure to ascend to its normal anatomical location during early embryonic development. This anomaly complicates traditional surgical approaches for renal calculi due to the kidney’s atypical positioning and associated anatomical challenges. We sought to summarize our experience with robotic-assisted pyelolithotomy (RPPK) for pelvic kidney stones. **Methods:** A retrospective review of patients who underwent RPPK was conducted between 2014 and 2023. Demographic data on stone characteristics, operative details, and postoperative outcomes were analyzed. **Results:** Four patients (three male; mean age—51.2 years with a range of 45–54; mean BMI—26.6 with a range of 22.3–32.2) underwent RPPK. All had a right-sided pelvic kidney; two had prior failed ureteroscopy. The mean stone diameter was 27.7 mm (range: 17–35); the mean density was 1207.5 HU (range: 905–1500). The mean operative time was 265 min (range: 200–323); the time from incision to closure was 142.2 min (range: 95–225). No ureteral stents or drains were used. Blood loss was negligible. The mean hospital stay was 2.2 days. No immediate complications were recorded. The mean follow-up was 11.75 months (range: 3–30). **Conclusions:** RPPK is safe and effective for managing pelvic kidney stones. Challenging renal anatomy stone size and density are not obstacles to the robotic approach unlike other fragmentation methods

## 1. Introduction

Pelvic kidney is a congenital anomaly characterized by the kidney’s failure to ascend to its normal anatomical location during early embryonic development, typically between the sixth and ninth weeks of gestation. This condition has an estimated prevalence of 1 in 3000 births [1]. The abnormal positioning of the kidney in the pelvis, rather than the abdomen, results in unique structural anomalies that can complicate both its function and the surgical approaches required for treatment [2].

The anatomical peculiarities of pelvic kidneys often include malrotation, where the renal pelvis faces anteriorly, and aberrant vascular structures that can obstruct the renal calyces or upper ureter [3]. These anomalies predispose individuals to chronic urinary obstruction and the formation of large renal calculi [4]. The presence of intestinal loops surrounding the pelvic kidney further complicates percutaneous access, making traditional surgical methods like percutaneous nephrolithotomy (PCNL) challenging [5]. Additionally, the angles required for flexible ureteroscopy are often suboptimal, hindering effective stone fragmentation and removal. The high density of stones in pelvic kidneys further complicates mechanical fragmentation via lasers, shock waves, or ultrasound [6].

Pelvic kidneys can also lead to other complications such as urinary tract infections (UTIs), hydronephrosis (swelling of the kidney due to urine buildup), and the increased risk of kidney trauma due to their lower position in the body. These complications can result in symptoms like abdominal or back pain, hematuria (blood in the urine), and high blood pressure [7].

The introduction of the da Vinci robotic system has revolutionized urological surgery, offering enhanced dexterity, precision, and visualization. These advantages are particularly beneficial in managing complex cases involving anatomically anomalous kidneys, such as pelvic kidneys [8]. The robotic system allows for greater maneuverability and control, which is crucial when dealing with the intricate anatomy and challenging positions of pelvic kidneys. Despite these advancements, studies on robotic-assisted pyelolithotomy (RPPK) for pelvic kidney stones remain scarce, with only a few reported cases to date [9].

Robotic pyelolithotomy has shown promising outcomes in terms of stone clearance rates, reduced blood loss, and shorter hospital stays compared to traditional methods [10]. A systematic review and meta-analysis reported an average operative time of 168.10 min, a hospital stay of 2.63 days, and a stone clearance rate of 87% [11]. The incidence of minor postoperative complications was 23.7%, while major complications were relatively low at 7% [12].

Robotic pyeloplasty, a procedure used to treat ureteropelvic junction (UPJ) obstruction, has demonstrated high success rates and improved patient outcomes compared to open surgery [13]. This minimally invasive technique reduces the risk of complications, shortens hospital stays, and allows for quicker recovery times [14]. The use of robotic technology in pyeloplasty and other urological procedures continues to evolve, offering new possibilities for the treatment of pelvic kidney anomalies [15].

This study aims to summarize our experience with RPPK for the management of pelvic kidney stones, highlighting the safety and efficacy of this approach. By providing a detailed analysis of our surgical outcomes, we hope to contribute valuable insights to the limited body of literature on this topic and support the broader adoption of robotic techniques in urological surgery [16].

## 2. Materials and Methods

### 2.1. Study Design

This study was a retrospective review of patients who underwent robotic-assisted pyelolithotomy (RPPK) for pelvic kidney stones at our institution between 2014 and 2023. All procedures were performed by a single surgeon with over 10 years of experience in robotic surgery and extensive expertise in stone management. Institutional Helsinki Committee approval was obtained prior to data collection.

### 2.2. Patient Selection

Patients included in this study were those diagnosed with pelvic kidney stones and who underwent RPPK. Demographic data, including gender, age, body mass index (BMI), side of surgery, and American Society of Anesthesiologists (ASA) physical status classification, were collected. Previous surgical history, particularly prior failed ureteroscopy and laser lithotripsy attempts, was also documented.

#### Inclusion Criteria

During the study period from 2014 to 2023, there were no other cases of pelvic kidneys with stones in our department. Therefore, all relevant cases were included in our study, and no other cases were managed with different treatment modalities.

### 2.3. Preoperative Assessment

All patients underwent non-contrast stone protocol computed tomography (NCCT) to assess stone size and density. The mean stone diameter and density were recorded to evaluate the complexity of the cases. Additionally, renal function was assessed using serum creatinine and estimated glomerular filtration rate (eGFR) measurements.

### 2.4. Surgical Technique

RPPK was performed using the da Vinci^®^ Si robotic system.

#### 2.4.1. Patient Positioning

Patients were placed in a 30-degree Trendelenburg position after standard preparation and draping. This position helps to optimize the surgical field by displacing the intestines away from the pelvic kidney.

#### 2.4.2. Port Placement

**Figure 1:**
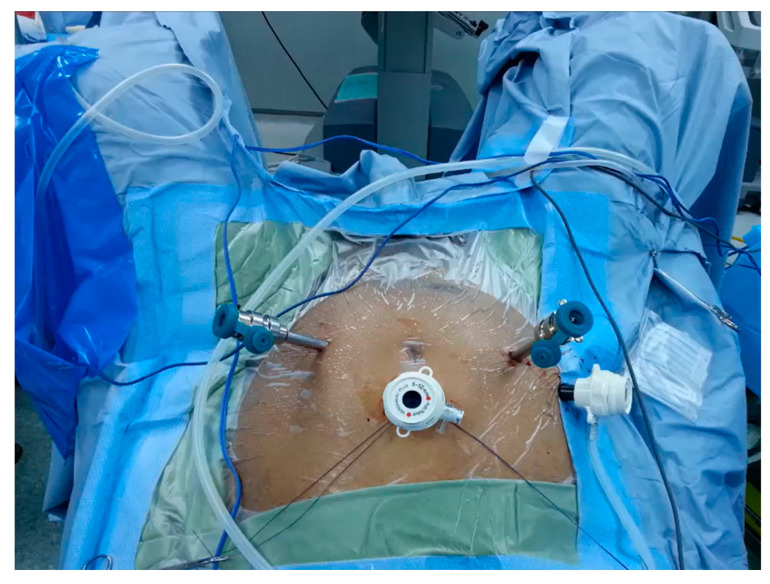
Surgical position and placement of working ports for robot-assisted stone extraction from a pelvic kidney using the da Vinci system.

Port placement was optimized for each patient, with specific attention to the anatomical variations of the pelvic kidney. The typical port configuration included three robotic ports and one assistant port, as illustrated in Figure 1. The ports were strategically placed to allow for maximum maneuverability of the robotic instruments.

#### 2.4.3. Surgical Approach

A transperitoneal approach was utilized. The pelvic kidney was identified, and the overlying peritoneum was opened. Dissection of the perinephric fat was performed to expose the renal pelvis. Care was taken to avoid injury to surrounding structures, such as the intestines and major blood vessels.

#### 2.4.4. Stone Removal

The renal pelvis was incised to locate and remove the stone. Intraoperative ultrasound was used in selected cases to accurately identify the renal pelvis and guide the incision. This technique ensured precise localization of the stones, minimizing unnecessary dissection.

#### 2.4.5. Closure

Layered closure of the renal pelvis was performed using a 26 mm needle and 3/0 V-Loc™ suture (Covidien, Dublin, Ireland). No ureteral stents or pelvic drains were placed post-surgery. Hemostasis was meticulously achieved to prevent postoperative bleeding.

### 2.5. Intraoperative and Postoperative Data

Operative times, including total operating room time and time from first incision to closure, were recorded. Estimated blood loss was measured using standard intraoperative techniques. The length of hospital stay was documented, and immediate postoperative complications were assessed using the Clavien–Dindo classification system. Postoperative pain was managed according to institutional protocols, and patients were encouraged to ambulate early to reduce the risk of thromboembolic events.

### 2.6. Follow-Up

Patients were followed up for a mean period of 11.75 months (range: 3–30 months). Follow-up assessments included clinical evaluations and imaging studies to monitor for stone recurrence and other complications. Imaging modalities used during follow-up included ultrasound and NCCT, depending on the clinical scenario.

### 2.7. Statistical Analysis

Descriptive statistics were used to summarize the data. Continuous variables were presented as means with ranges, while categorical variables were presented as frequencies and percentages. Statistical analyses were performed using SPSS software (version 25.0; IBM Corp., Armonk, NY, USA). A *p*-value of <0.05 was considered statistically significant.

## 3. Results

### 3.1. Patient Demographics and Stone Characteristics

**Figure 2:**
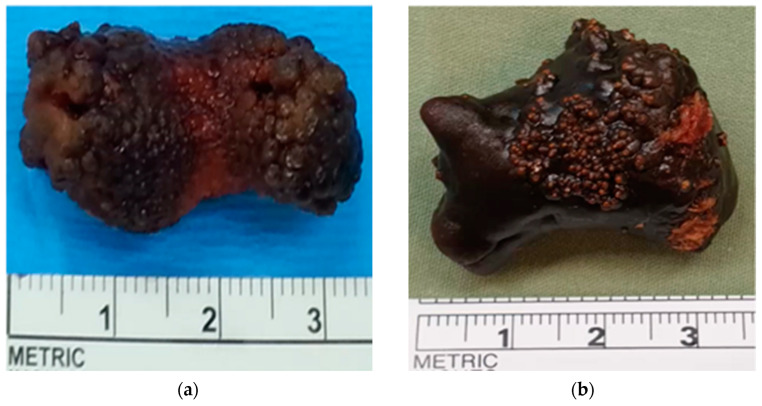
Stones extracted from two patients with pelvic kidneys following robotic-assisted pyelolithotomy. (**a**) Stone extracted from patient 1; (**b**) large stone extracted from patient 2.

Four patients (three male and one female; mean age of 51.2 years with a range of 45–54; mean BMI of 26.6 with a range of 22.3–32.2) underwent RPPK. All patients had a right-sided pelvic kidney. Two patients had prior failed ureteroscopy and laser lithotripsy attempts. The mean stone diameter was 27.7 mm (range: 17–35 mm) and the mean stone density was 1207.5 HU (range: 905–1500 HU) (shown in Figure 2 for cases 1–2) Demographic and perioperative data are summarized in Table 1.

### 3.2. Operative Details

The mean operative time was 265 min (range: 200–323 min), with a mean time from incision to closure of 142.2 min (range: 95–225 min). No ureteral stents or pelvic drains were used. Blood loss was negligible in all cases, consistent with findings from other studies that report minimal intraoperative bleeding during robotic pyelolithotomy [1,2].

### 3.3. Postoperative Outcomes

The mean hospital stay was 2.2 days (range: 1–4 days), which aligns with other reports indicating a short postoperative recovery period for robotic-assisted procedures [3]. No immediate complications were recorded within the first week or month post-surgery. The average follow-up period was 11.75 months (range: 3–30 months). During this period, no patients experienced stone recurrence or significant postoperative complications.

### 3.4. Stone Clearance and Efficacy

All patients achieved complete stone clearance, as confirmed by postoperative imaging. This 100% stone-free rate is higher than the rates reported for other modalities such as PCNL and ureteroscopy, which often face challenges due to the anatomical complexities of pelvic kidneys [4]. The high stone clearance rate observed in this study is consistent with the efficacy of robotic pyelolithotomy in managing complex renal stones [5].

### 3.5. Comparative Analysis

Compared to traditional methods like PCNL and ureteroscopy, RPPK demonstrated several advantages, including reduced operative time, minimal blood loss, and shorter hospital stays. The robotic approach also allowed for precise dissection and stone removal, minimizing the risk of injury to surrounding structures. These findings support the growing body of evidence that robotic-assisted techniques are effective and safe for managing complex renal stones, particularly in anatomically challenging cases.

## 4. Discussion

### 4.1. Comparison with Previous Studies

Pelvic kidneys pose a unique surgical challenge due to their atypical anatomy. Located in the pelvis in a malrotated position with the renal pelvis usually facing anteriorly, they often feature impaired urine drainage, highly inserted ureters, and abnormally positioned blood vessels that may obstruct renal calyces or the upper ureter [3,4]. This anatomical configuration creates an environment conducive to stone formation.

Our study demonstrates that robotic-assisted pyelolithotomy (RPPK) is a safe and effective method for managing pelvic kidney stones, achieving a 100% stone-free rate. This is notably higher than the stone-free rates reported for other modalities such as percutaneous nephrolithotomy (PCNL) and ureteroscopy, which often face challenges due to the anatomical complexities of pelvic kidneys [1,2]. Previous studies have shown that PCNL, while effective, can be associated with significant complications, including bleeding, infection, and injury to surrounding organs [3]. In contrast, RPPK offers a minimally invasive alternative with reduced operative times, minimal blood loss, and shorter hospital stays [4].

The American Urological Association guidelines suggest robotic surgery as a first-line treatment for complex kidney stones or when concurrent upper urinary tract reconstruction is needed [9].

### 4.2. Advantages of Robotic-Assisted Techniques

The advantages of robotic-assisted techniques in urological surgery are well documented. The da Vinci robotic system provides enhanced dexterity, precision, and visualization, which are crucial when dealing with the intricate anatomy of pelvic kidneys. Our findings align with other studies reporting high success rates and low complication rates for robotic-assisted pyelolithotomy [5,6]. The ability to perform precise dissection and stone removal minimizes the risk of injury to surrounding structures, a significant advantage over traditional methods [7].

### 4.3. Clinical Implications

The clinical implications of our findings are significant. The high stone clearance rate and low complication rate associated with RPPK suggest it should be considered a viable first-line treatment option for patients with large pelvic kidney stones. This is particularly relevant for patients who have failed previous treatments such as ureteroscopy and laser lithotripsy. The minimally invasive nature of RPPK also means shorter recovery times and reduced hospital stays, leading to improved patient satisfaction and reduced healthcare costs [8].

#### 4.3.1. Comparison with PCNL

Percutaneous nephrolithotomy (PCNL) is widely regarded as the gold standard for the treatment of large renal stones, particularly those larger than 2 cm. However, its application in anatomically challenging cases, such as pelvic kidneys, presents unique difficulties. This section aims to provide a balanced comparison between PCNL and RPPK, highlighting their respective advantages and limitations.

#### 4.3.2. Efficacy and Stone-Free Rates

PCNL is known for its high stone-free rates (SFRs), often exceeding 85% in standard cases [10]. However, the anatomical complexities of pelvic kidneys can reduce the efficacy of PCNL. The presence of aberrant vascular structures and the malrotated position of the kidney can impede access and stone clearance. In contrast, RPPK has demonstrated a 100% stone-free rate in our study, suggesting superior efficacy in these challenging cases.

#### 4.3.3. Complications and Safety of PCNL

While effective, PCNL is associated with significant complications, including bleeding, infection, and injury to surrounding organs [11]. The risk of severe complications is heightened in patients with pelvic kidneys due to the kidney’s atypical positioning and the proximity of major blood vessels. RPPK, on the other hand, offers a minimally invasive alternative with reduced operative times, minimal blood loss, and shorter hospital stays. The enhanced dexterity and precision of the da Vinci robotic system allow for meticulous dissection and stone removal, minimizing the risk of injury to surrounding structures [12].

#### 4.3.4. Operative Time and Hospital Stay

Studies have shown that the operative time for PCNL can be longer in cases involving pelvic kidneys due to the need for careful navigation around anatomical obstacles [13]. Our study found that the mean operative time for RPPK was 265 min, which is comparable to or shorter than the operative times reported for PCNL in similar cases. Additionally, the mean hospital stay for RPPK patients was 2.2 days, significantly shorter than the typical hospital stay for PCNL patients, which can range from 3 to 7 days.

#### 4.3.5. Economic Considerations

The cost of robotic surgery is often cited as a limitation due to the high initial investment in robotic systems like the da Vinci system. However, the reduced hospital stays and lower complication rates associated with RPPK may offset these costs over time. An economic analysis considering these factors would provide valuable insights into the feasibility and broader adoption of RPPK.

#### 4.3.6. Patient Selection and Anatomical Considerations

PCNL remains a viable option for many patients, particularly those with standard renal anatomy. However, in cases involving pelvic kidneys, the anatomical challenges can make PCNL less safe and effective. The decision to use RPPK should be based on a thorough preoperative assessment, including detailed imaging studies to map the renal anatomy and identify potential obstacles.

#### 4.3.7. Literature Review and Systematic Comparisons

Several systematic reviews and meta-analyses have compared PCNL with other treatment modalities. For instance, a study by Bai et al. [17] found that while PCNL had higher initial SFRs, the overall complication rates were also higher compared to retrograde intrarenal surgery (RIRS). Similarly, a meta-analysis by Qiu et al. [18] highlighted the higher complication rates and longer hospital stays associated with PCNL in obese patients. These findings underscore the need for careful patient selection and the potential benefits of RPPK in anatomically complex cases.

### 4.4. Limitations

Despite the promising results, our study has several limitations that need to be addressed. They are as follows:

- Small sample size: Our study was based on a small sample size (n = 4), which limits the generalizability of our findings. Larger multicenter studies are needed to validate our results and provide more robust evidence for the efficacy and safety of RPPK. Future research should aim to include a more diverse and larger patient population to strengthen the findings.
- Single-center study: The study was conducted at a single institution, which may limit the generalizability of the findings. Multicenter studies could provide more diverse data and enhance the external validity of the results.
- Selection bias: Given the retrospective nature of the study, there was a potential for selection bias. Patients who underwent RPPK might have been selected based on specific criteria that are not representative of the broader population with pelvic kidney stones.
- Lack of a control group: The study did not include a control group for comparison. Including a control group undergoing alternative treatments would provide a more robust comparison of the efficacy and safety of RPPK.
- Short follow-up period: Although the mean follow-up period was 11.75 months, this may still be insufficient to fully assess long-term outcomes and complications. We did not include structured long-term follow-up data on recurrence rates and postoperative complications. This limits our understanding of the long-term efficacy and safety of RPPK. Future studies should incorporate long-term follow-up protocols to monitor patients over an extended period.
- Technological dependence: The study relied heavily on the use of the da Vinci robotic system, which may not be available in all healthcare settings. This limits the applicability of the findings to institutions with access to such advanced technology.
- Operator experience: The outcomes of robotic-assisted surgeries can be highly dependent on the surgeon’s experience and proficiency with the robotic system. Variability in operator skill could influence the results, and this factor should be considered when interpreting the findings.
- Retrospective nature: The retrospective design of our study introduced potential selection bias and other inherent limitations. This design restricted our ability to control for all confounding variables. Prospective studies are recommended to provide more robust and unbiased data.
- Economic considerations: Our study did not include an economic analysis of the feasibility and broader adoption of the da Vinci system. Given the variability in the availability of this technology across healthcare facilities, an economic evaluation would provide valuable insights. Future studies should assess the cost-effectiveness of RPPK to support its wider implementation.
- Postoperative management without stents or drains: We chose not to use stents or drains postoperatively, but our study lacked a detailed justification for this approach. Future research should include a literature review on protocols involving stents or drains and provide a rationale for the chosen postoperative management strategy.

In conclusion, while our study provided valuable insights into the efficacy and safety of RPPK, addressing these limitations in future research will enhance the validity and applicability of our findings.

### 4.5. Future Directions

Future research should focus on larger prospective studies to confirm the benefits of RPPK for pelvic kidney stones. It would also be valuable to compare RPPK directly with other treatment modalities in randomized controlled trials to provide higher-level evidence for its efficacy and safety. Additionally, further studies could explore the cost-effectiveness of RPPK compared to other treatments, as well as its long-term outcomes and impact on patient quality of life [10].

### 4.6. Additional Insights from Recent Studies

Recent systematic reviews and meta-analyses have highlighted the growing interest in robotic pyelolithotomy as an alternative to PCNL for managing complex renal stones. A meta-analysis by Wang et al. reported an 87% stone clearance rate for robotic pyelolithotomy, with a mean operative time of 168.10 min and a mean hospital stay of 2.63 days [11]. These findings are consistent with our results, further supporting the efficacy and safety of robotic-assisted techniques.

Moreover, the incidence of minor postoperative complications (Clavien grade I–II) was reported to be 23.7%, and major complications (Clavien grade ≥ III) were 7% [12]. These complication rates are comparable to those reported for PCNL, but with the added benefits of reduced blood loss and shorter recovery times. The ability to manage large and complex stones with minimal invasiveness makes robotic pyelolithotomy an attractive option for both patients and surgeons [13].

### 4.7. Technological Advancements and Training

The continuous advancements in robotic technology and the increasing experience of surgeons are likely to further improve the outcomes of robotic-assisted procedures. The da Vinci system’s enhanced capabilities, such as tremor filtration and 3D visualization, allow for more precise and controlled surgical maneuvers. As more urological surgeons become proficient in robotic techniques, the accessibility and utilization of robotic pyelolithotomy are expected to increase [14].

Training and proficiency in robotic surgery are crucial for optimizing patient outcomes. Studies have shown that the learning curve for robotic-assisted pyelolithotomy is relatively short, with significant improvements in operative times and complication rates observed after the initial cases. This suggests that with adequate training and experience, surgeons can achieve high success rates and low complication rates, making robotic pyelolithotomy a feasible option for a wider range of patients [15].

Various approaches for treating stones in pelvic kidneys have been described. A comprehensive systematic review summarizes all relevant studies on this topic up to 2020 [1].

A comprehensive review of 23 studies from 1993 to 2019, encompassing 119 patients, compared different treatment modalities.

The methodologies investigated encompassed a range of interventions, including extracorporeal shock wave lithotripsy (ESWL), ureteroscopy (URS), PCNL, a combined approach of laparoscopic surgery with PCNL, standalone laparoscopy, and robot-assisted stone extraction.

The stone-free rate (SFR) was defined as the percentage of cases in which no residual stones requiring further treatment were found after the first treatment.

According to the mentioned systematic review [1], the SFR varied widely: ESWL showed the lowest SFR (25–66%), followed by URS (66–75%) and PCNL (85%).

The main challenges with these methods include difficulty in fragment clearance and limited stone access due to challenging angles. Moreover, the high density of stones, as presented in our series, complicates mechanical fragmentation by laser, shock waves, or ultrasound.

Additionally, the problematic access to the pelvic kidney due to surrounding intestinal loops prevents safe percutaneous access to the renal pelvis.

The introduction of the da Vinci robotic system has revolutionized the surgical approach to kidney stones, especially in complex cases in treating large stones in non-pelvic kidneys, particularly when concurrent reconstructive procedures of the upper urinary tract, such as pyeloplasty, are required [2,3,8].

Its advantages include dexterity in seven degrees of freedom, excellent visualization, tremor cancellation, and precise instrument control. These features have led to reported 100% success rates in pelvic kidney stone removal, both in our series and others [4,5,6].

Despite the robot’s advantages, it is crucial to emphasize the necessity of high-quality preoperative imaging, such as CT, to identify the renal artery crossing and ureteral location, thereby avoiding intraoperative injury.

In addition to superior imaging, intraoperative ultrasound may be required for precise incision localization, as observed in two of our cases whereby the renal pelvis was oriented posteromedially rather than anteriorly with highly adherent and edematous perinephric fat as an indicative marker of chronic inflammation. This anatomical configuration complicated the identification of the appropriate incision site and necessitated, as mentioned, intraoperative imaging to avoid inadvertent injury to the ureter, major blood vessels, or massive hemorrhage resulting from incision into the renal parenchyma itself.

Although the estimated prevalence of pelvic kidneys is 1 in 3000 births [1,4], and despite the high potential for stone formation due to their unique structure, the incidence of stones in pelvic kidneys is low, resulting in few reports on their treatment.

A literature review revealed only three prior reports of robotic-assisted stone removal from pelvic kidneys [4,5,6] (Table 2).

In summary of all cases, a clear male predominance was observed. All patients were operated on in their fifth to sixth decades of life, with similar outcomes regarding operative times, blood loss, and length of hospitalization. Consistent with our study, no complications were reported by others.

In the current study, the substantial size of the stones, even relative to other cases, and their high density are noteworthy. These two factors justified the use of robotic assistance for stone removal.

Nayyar et al. [4] described leaving a ureteral stent at the procedure’s conclusion, while Antonelli et al. [6] reported leaving a pelvic drain post-surgery. However, in our experience, when using a barbed suture such as V-Loc and achieving good closure of the renal pelvis followed by the proper approximation of perinephric fat and peritoneal edges over the operated kidney, there is no advantage to leaving a ureteral stent or drain. This approach reduces patient discomfort and minimizes the risk of postoperative infections.

The main limitations of this study are the small sample size and the lack of a long-term follow-up. However, the absence of patient return suggests no stone recurrence.

The American Urological Association guidelines suggest robotic surgery as a first-line treatment for complex kidney stones or when concurrent upper urinary tract reconstruction is needed [16]. Given the high success rates in pelvic kidney stone removal, we concur that this approach can be offered as a first-line treatment, even without the need for urinary tract reconstruction [2].

## 5. Conclusions

Robotic-assisted pyelolithotomy (RPPK) has proven to be a highly effective and safe method for managing pelvic kidney stones, as demonstrated by our study’s 100% stone-free rate. This technique offers several advantages over traditional methods, such as percutaneous nephrolithotomy (PCNL) and ureteroscopy, including reduced operative times, minimal blood loss, and shorter hospital stays. The enhanced dexterity, precision, and visualization provided by the da Vinci robotic system are particularly beneficial when dealing with the complex anatomy of pelvic kidneys.

Our findings suggest that RPPK should be considered a viable first-line treatment option for patients with large pelvic kidney stones, especially those who have failed previous treatments.

Despite the promising results, our study’s limitations, including the small sample size and retrospective design, highlight the need for larger prospective studies to validate our findings. Future research should focus on comparing RPPK with other treatment modalities in randomized controlled trials, exploring the cost-effectiveness of this technique, and assessing long-term outcomes and quality of life.

## Figures and Tables

**Table 1:** Demographic and perioperative data of patients. HUs = Hounsfield units.

Patient Number	Sex	Age	ASA	BMI	Side	Maximum Stone Diameter (mm)	Stone Density (HU)	Operating Room Time (min)	Incision to Closure Time (min)	Blood Loss (mL)	Length of Stay (Days)	Follow-Up Duration (Months)
1	Female	53	2	26.6	Right	25	1300	200	95	10	3	4
2	Male	45	1	32.2	Right	34	905	320	119	20	4	10
3	Male	53	2	25.4	Right	35	1500	323	225	0	1	30
4	Male	54	1	22.3	Right	17	1125	216	130	20	1	3

**Table 2:** Reported cases in the literature of stone extraction from pelvic kidneys using the da Vinci robot.

Author	Age	Sex	Side	Maximum Stone Diameter (mm)	Console Time (min)	Blood Loss (mL)	Length of Stay (Days)
Nayyar et al. [4]	55	Male	Left	13	56	-	2
Al-Yousef et al. [5]	46	Male	Left	12	150	<100	2
Antonelli et al. [6]	44	Male	Bilateral	17, 12	190	50	4

## Data Availability

Data available upon request to correspondence.
